# Excellent Room-Temperature NO_2_ Gas-Sensing Properties of TiO_2_-SnO_2_ Composite Thin Films Under Light Activation

**DOI:** 10.3390/nano15110871

**Published:** 2025-06-05

**Authors:** Victor V. Petrov, Aleksandra P. Starnikova, Maria G. Volkova, Soslan A. Khubezhov, Ilya V. Pankov, Ekaterina M. Bayan

**Affiliations:** 1 Institute of Nanotechnologies, Electronics, and Equipment Engineering, Southern Federal University, 347928 Taganrog, Russia; a.starnikova@mail.ru; 2 Department of Chemistry, Southern Federal University, 344090 Rostov-on-Don, Russia; mvol@sfedu.ru (M.G.V.); ekbayan@sfedu.ru (E.M.B.); 3 Qingdao Innovation and Development Center, Harbin Engineering University, Qingdao 266000, China; soslan.khubezhov@gmail.com; 4 Institute of Physical and Organic Chemistry, Southern Federal University, Stachki Av. 194/2, 344090 Rostov-on-Don, Russia; ipankov@sfedu.ru

**Keywords:** gas sensors, light activation, metal oxide, composites, thin films, TiO_2_, SnO_2_

## Abstract

Thin TiO_2_–SnO_2_ nanocomposite films with high gas sensitivity to NO_2_ were synthesized by oxidative pyrolysis and comprehensively studied. The composite structure and quantitative composition of the obtained film nanomaterials have been confirmed by X-ray photoelectron spectroscopy, high-resolution transmission electron microscopy, and energy dispersive X-ray spectroscopy, which causes the presence of n-n heterojunctions and provides improved gas-sensitive properties. The sensor based on the 3TiO_2_–97SnO_2_ film has the maximum responses, which is explained by the existence of a strong surface electric field formed by large surface potentials in the region of TiO_2_–SnO_2_ heterojunctions detected by the Kelvin probe force microscopy method. Exposure to low-intensity radiation (no higher than 0.2 mW/cm^2^, radiation wavelength—400 nm) leads to a 30% increase in the sensor response relative to 7.7 ppm NO_2_ at an operating temperature of 200 °C and a humidity of 60% RH. At room temperature (20 °C), under humidity conditions, the response is 1.8 when exposed to 0.2 ppm NO_2_ and 85 when exposed to 7.7 ppm. The lower sensitivity limit is 0.2 ppm NO_2_. The temporal stability of the proposed sensors has been experimentally confirmed.

## 1. Introduction

Gas sensors based on metal oxide semiconductors (MOSs) are widely used for air monitoring, in the control of toxic gases’ content, and in the non-invasive diagnosis of various human diseases [1,2,3]. The MOS films used in gas sensors have excellent functional characteristics (high sensitivity, fast response, and selectivity), chemical inertness to the environment, cheapness, and ease of synthesis. The disadvantages of pure MOSs are their high operating temperature and low selectivity [4,5]. There are many methods to enhance MOS characteristics, including refined synthesis methods, obtaining nanostructures, modifying and creating composites, and light activation [6,7,8]. The most widely used MOSs are SnO_2_, TiO_2_, In_2_O_3_, ZnO, CuO, and WO_3_ [9]. Tin dioxide, a wide-band n-type semiconductor, is most often used as a gas-sensitive material [10]. At the same time, composites based on it are used to improve the functional properties of SnO_2_ [11]. As modifying agents, n-type semiconductor metal oxides such as In_2_O_3_ [12], ZnO [13], TiO_2_ [14], and others can be used. Composites based on SnO_2_ and TiO_2_ are the most promising for improving gas-sensitive properties due to the same metal ion charge, the close ionic radius of the metal, and the same type of crystallization in the rutile phase. The band gap of SnO_2_ (3.5–3.6 eV) is higher than that of TiO_2_ (3.0 eV for rutile). At the same time, the bottom of the conduction band of SnO_2_ (−0.05 eV) is located lower than that of TiO_2_ (−0.24 eV). As a result, in SnO_2_/TiO_2_ heterostructures, the interaction of SnO_2_ and TiO_2_ forms a type-II heterojunction in which the top of the TiO_2_ valence band is located in the band gap of SnO_2_, and the bottom of the TiO_2_ conduction band is located above the bottom of the SnO_2_ conduction band [15].

Consequently, a contact potential difference (potential barrier) is established between the TiO_2_ and SnO_2_ structures, facilitating electron transfer from the TiO_2_ conduction band to the SnO_2_ conduction band. At the same time, the adsorption of oxygen molecules (O_2_) on the surface of SnO_2_ and TiO_2_ grains is enhanced due to the existence of a potential barrier. This effect works well in gas sensors based on two-layer TiO_2_/SnO_2_ structures. In one case, the sensor showed a response of 696 to 400 ppb NO_2_ at an operating temperature of 123 °C [16]. In another study, the sensor demonstrated a response of ~4500 at 150 °C for 4 ppm NO_2_ [17]. The influence of n-n TiO_2_-SnO_2_ heterojunctions is manifested in the formation of TiO_2_ nanoparticles on the surface of SnO_2_ nanosheets. A sensor based on them demonstrated a response equal to 52.3 by exposure to 100 ppm triethylamine at an operating temperature of 260 °C [18]. It was also shown that the SnO_2_-TiO_2_ core–shell heterostructures exhibited highly gas-sensitive properties: 65.08% in relation to 50 ppm NO_2_ at an operating temperature of 100 °C [19].

However, TiO_2_-SnO_2_ composite nanostructures are most often used in the study of the catalytic oxidation processes of VOGs [20]. Studies of their gas-sensing characteristics are rarely investigated. Thus, in [21], it was shown that a nanocomposite film based on SnO_2_ with the addition of 5% TiO_2_ leads to an increase in the number of chemisorption centers of gas molecules, and, accordingly, to an increase in the response (≈4) of the sensor compared to pure tin dioxide [22] when exposed to 10,000 ppm H_2_ at an operating temperature of 400 °C. However, the particle sizes in these studies were above 50 nm, although it is known that the best gas-sensing properties are shown by materials whose particle sizes do not exceed 30 nm [23].

In recent years, more and more attention has been paid to the development of light-activated gas sensors to reduce the operating temperature, as well as to increase selectivity and sensitivity [16]. UV [24,25] and visible activation [6,26] are used for this purpose. A gas sensor based on a gas-sensitive material with TiO_2_@SnO_2_ nanospheres demonstrates high sensitivity (20) and excellent selectivity for 10 ppm formaldehyde under UV activation at room temperature (RT) [27]. The response and recovery times were 20 and 56 s, respectively, which is much shorter than those of pure TiO_2_ (52 and 164 s).

A sensor based on a two-layer TiO_2_/SnO_2_ film with layer thicknesses of 20 nm TiO_2_ and 200 nm SnO_2_, activated by UV radiation, showed high activity against O_2_ at a low operating temperature (92 °C) [28]. The sensor also demonstrates good long-term stability, selectivity, and resistance to humidity.

The activating effect of UV radiation is explained by the fact that its impact causes the desorption of oxygen molecules. As is known, at temperatures up to 150–200 °C, metal oxides contain adsorbed oxygen molecules and ions (O_2_ and O_2_^−^), and at temperatures above 200 °C, they contain atomic oxygen ions (O^2−^ and O^−^) [29,30,31]. The temperature limit of the dissociation process of molecular oxygen ions is in the range of 160–200 °C [6,32]. Upon photoexcitation by UV and visible activation, nonequilibrium (photogenerated) charge carriers (electrons (e^−^) and holes (p^+^)) appear in the semiconductor metal oxide. The holes react with the adsorbed oxygen ions (O^2−^) and desorb them from the surface (1):

(1) p^+^ + O_2_^−^ → O_2_(gas)

Oxygen molecules can react with photoinduced electrons to generate additional photoinduced oxygen ions (2) [33]:

(2) O_2_(gas) + e^−^ → O_2_^−^

For n-type semiconductors, the first process leads to the gas-sensitive material conductivity increasing, and the second leads to it decreasing. Since the n-type semiconductor’s conductivity increases, the reaction (1) is more likely.

The highest response value (about 5.9) is observed at a low radiation intensity (1 mW/cm^2^) [34]. The authors explained that radiation shifts the balance towards the O_2_ molecules’ desorption rate.

The authors of [35] showed the possibility of achieving high sensitivity in UV-activated (220 nm), low-intensity (150 µW/cm^2^) gas sensors based on a porous SnO_2_@TiO_2_ heterostructure operating at room temperature. The response to 50 ppm CO was 56.7%, and the lower detection limit (LOD) was 1 ppm. However, an increase in air humidity from 0% to 80% led to a decrease in the response value by almost 4.5 times. Water molecules and OH^−^_ads_ groups occupy surface adsorption centers, preventing the gas molecules’ adsorption [29,36,37]. It was also shown [38,39] that when exposed to UV radiation, the effect of temperature drift is not so noticeable, since the concentration of photogenerated charge carriers is comparable to the charge carrier concentration generated as a result of gas molecules’ sorption on the gas-sensitive material’s surface. It was also shown that the radiation power should not exceed 1 mW/cm^2^, since high radiation power can lead not only to the photogeneration of a significant number of charge carriers, but also to the gas molecule’s dissociation even before its interaction with a gas-sensitive material.

According to other works, the prospect of using photoactivation by light of tin dioxide-based composites has been established. We have previously shown that a new approach to the synthesis of composites using oxidative pyrolysis makes it possible to obtain films with good gas-sensitive characteristics [40,41]. The review presents a significant number of NO_2_ sensors based on various gas-sensitive materials; however, sensors based on TiO_2_–SnO_2_ films are not described [42]. The most sensitive NO_2_ sensor based on a two-layer porous TiO_2_/SnO_2_ structure was described this year [43]. The sensor detects NO_2_ with a concentration of up to 4 ppb (response 0.6%) and 10 ppb (response 1.3%) at room temperature and exposure to UV radiation (365 nm) of minimum intensity (3 μW/cm^2^). The high sensitivity is explained by the porous matrix structure of the gas-sensitive material. The disadvantages of the sensor, in our opinion, are the complex two-stage method of forming a two-layer porous TiO_2_/SnO_2_ structure, as well as the fact that the measuring electrodes are formed from aluminum. Aluminum, as is known, is a chemically active material subject to active oxidation in a humid environment. The method of forming the TiO_2_/SnO_2_ structure used by the authors does not allow one to use the standard sensor platforms for manufacturing a sensor with platinum electrodes.

As can be seen from the above analysis, there are not many descriptions of nitrogen dioxide sensors based on composite nanosized TiO_2_–SnO_2_ films in the literature. Therefore, the oxidative pyrolysis synthesis method was also used to obtain TiO_2_–SnO_2_ films in this study. The most common toxic gas, nitrogen dioxide, was used as a model gas for the study. Studies were also conducted on ethanol vapor, methane, and carbon monoxide.

The aim of this work was to study the gas-sensitive properties of gas sensors based on TiO_2_–SnO_2_ composite films with the addition of 1, 3, or 5% TiO_2_ at RT and temperatures close to it when the NO_2_ concentration is 0.15–7.7 ppm, under low-intensity radiation (no higher than 0.2 mW/cm^2^). The novelty of this work lies in the use of a new technology to create gas-sensitive composite sensor films. These films are composed of TiO_2_ and SnO_2_ nanocrystals with dimensions of 10–20 nm, which create a high surface potential during contact. This high surface potential, when activated by light, provides excellent sensitivity to NO_2_ gas at room temperature. It is important that the work shows the possibility of measuring at 60% humidity, which makes it possible to measure under real conditions.

## 2. Materials and Methods

### 2.1. Chemicals and Substrates

The chemicals used in this study, such as tin (IV) chloride pentahydrate (SnCl_4_·5H_2_O), titanium butoxide ((C_4_H_9_O)_4_Ti), and the organic acid and 1,4-dioxane, were purchased from ECROS, Russia. The synthesis of the intermediate product used to obtain TiO_2_–SnO_2_ films was carried out by the oxidative pyrolysis of salts. The amounts of (SnCl_4_·5H_2_O) and ((C_4_H_9_O)_4_Ti) were calculated based on the ratios of Ti/Sn = 1:99, 3:97, and 5:95 mol. %. The synthesis procedure is described in more detail in the paper [44]. In this way, precursors were obtained for the further formation of films with the compositions 1TiO_2_–99SnO_2_, 3TiO_2_–97SnO_2_, and 5TiO_2_–95SnO_2_.

### 2.2. Gas Sensor Design

**Figure 1:**
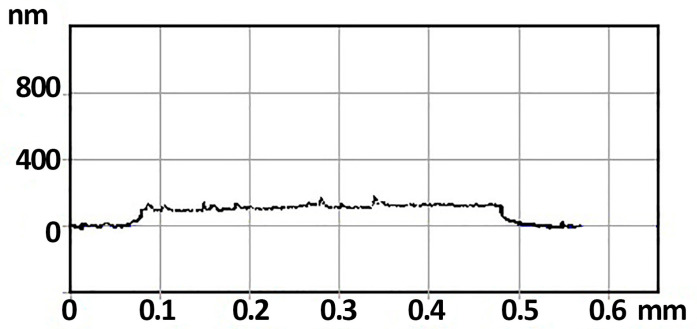
Profilometry of a 3TiO_2_–97SnO_2_ film on a sensor platform.

A 0.1 mm thick aluminum oxide sensor platform (1.6 × 1.6 mm^2^) was selected to form the gas sensor. A platinum thin-film heater with an electrical resistance of 15–20 Ω is formed on the non-working side of the sensor platform. On the working side of the substrate, a contact metallization is formed in the form of two counter-pin contacts with a distance of 100 μm between them. A precursor of the appropriate concentration was applied to a substrate and annealed at a temperature of 600 °C for two hours using a heater. Thus, the films 1TiO_2_–99SnO_2_, 3TiO_2_–97SnO_2_, and 5TiO_2_–95SnO_2_ were formed on the sensor platform. Figure 1 shows a profilogram (Alfa–Step D–100 contact stylus profiler (KLA–Tencor, Milpitas, CA, USA)) of 3TiO_2_–97SnO_2_; the film thickness is approximately 120 ± 20 nm.

### 2.3. Characterization of Thin Films

The surface composition of TiO_2_–SnO_2_ films obtained with different Ti/Sn ratios was studied by X-ray photoelectron spectroscopy. The research was carried out in an ultra-high vacuum installation K-Alpha ThermoScientific (Waltham, MA, USA) (2.4 × 10^−9^ mBar) with a source of monochromatic X-rays Al—Kα with quantum energy *hν* = 1486.6 eV.

The positions of the core photoelectronic lines of the reference samples took the values of Au 4f = 84 eV, Ag 3d = 368.2 eV, and Cu 2p = 932.6 eV, in this case, the position of the photoelectronic line for atmospheric carbon C 1s corresponded to the binding energy BE = 284.8 eV. The surface composition of the samples was determined from high-resolution spectra obtained in constant transmission energy mode (pass energy = 20 eV) with a spectral resolution of 0.1 eV, and the value of the statistical accumulation was (number of scans) N = 10.

Transmission electron microscopy (TEM), scanning transmission electron microscopy (STEM), and energy dispersive X-ray spectroscopy (EDX) using a Multi-purpose Electron Microscope JEM-F200 (JEOL, Akishima, Tokyo, Japan) operating at an accelerating voltage of 200 kV, equipped with a cold field emission electron gun and supported with a Bruker Xflash 6T/60 Quantax 400-STEM (JEOL, Akishima, Tokyo, Japan) were used to study the shape and size of crystallites and the elemental composition of the thin films. For both TEM and EDX measurements, a JEOL EM-01361RSTHB (Akishima, Tokyo, Japan) double-tilt beryllium specimen holder was used.

Optical transmission spectra were obtained using a Varian Cary-100 spectrophotometer (Beijing, China) in the range of 300–1000 nm. The band gap was determined using the Tauc plot by Equation (3):

(3) $${\left(\alpha h\nu \right)}^{2}=A(h\nu -E_{g})$$

where *h* is Planck’s constant, *A* is a constant, and *E_g_* is the optical band gap [45].

Assessment of the electrophysical properties of the obtained film samples was carried out on a software and hardware measuring complex that allows one to measure the effect of resistance on temperature dependence [13].

Gas-sensitive measurements were carried out on a stand with the possibility of activating gas-sensor interactions by UV–visible radiation with a wavelength of 400 nm (LED, GNL-3014VC, G-nor Electronics, Zhuangshi, China). The intensity of UV radiation did not exceed 0.2 mW/cm^2^.

The gas sensor’s response to the concentrations of 0.08–7.7 ppm NO_2_ balanced with synthetic air was measured at operating temperatures in the range of RT—200 °C. High-pressure balloons containing synthetic air and a mixture of synthetic air with test gas (LLC Hogas, Moscow, Russia) were employed as the primary sources. The gases were injected at a flow rate of 0.1–0.2 dm^3^/min using a gas-mixing generator (Microgaz F, Moscow, Russia) [13]. When measuring in humidity conditions of 60 ± 3%, a specially created stand was used, in which synthetic air was bubbled through deionized water. Humidity control was carried out using an HTTP.PF-U10 humidity sensor (KIP-Service LLC, Krasnodar, Russia).

Thus, gas sensitivity was measured under conditions of synthetic or humid air, or air and light activation, or humid air and radiation exposure. The sensors were initially exposed to the air or humid air, or air and light activation, or humid air and light activation for 60 min to stabilize the baseline resistance. The response of the sensor elements was calculated using Equation (4):

(4) S = R_g_(NO_2_)/R_0_,

where *R*_0_ is the sensor resistance in synthetic air (or humid synthetic air); *R_g_*(NO_2_) is the sensor resistance in the mixture of synthetic air (or humid synthetic air) and NO_2_. Subsequently, the gas sensor which exhibited the best gas-sensing performance was tested upon exposure to NO_2_.

## 3. Results and Discussion

### 3.1. Morphology, Structure, and Elemental Composition Analysis

**Figure 2:**
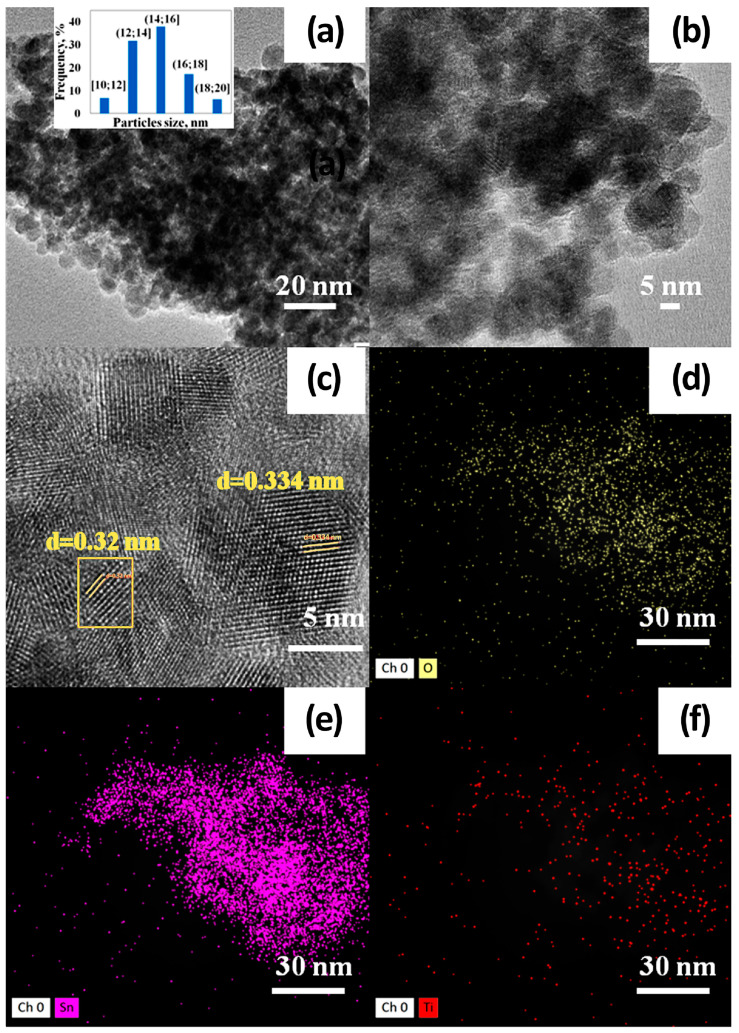
TEM images of 1TiO_2_–99SnO_2_ with different scale (**a**–**c**) and EDX analysis (**d**–**f**).

High-resolution TEM (HR TEM) has been used to study the morphology and crystal structure of synthesized nanomaterials. Examination of the 1TiO_2_–99SnO_2_ by TEM (Figure 2) showed that the obtained materials are nanostructured and consist of spherical particles measuring 12–16 nm (Figure 2a,b). The software Digimizer 6.4.5 was used to measure the particle size, and we estimated 200 nanoparticles to build the statistical distribution. It was shown that the main part of the nanoparticles (70%) are in the range of 12–16 nm, so the size is about 14 ± 2 nm. In total, 7, 17, and 6% of nanoparticles are in the ranges of 10–12, 16–18, and 18–20 nm, respectively. In previous studies [44,46], we analyzed the SEM images of 1TiO_2_–99SnO_2_, 5TiO_2_–95SnO_2_, and 50TiO_2_–50SnO_2_ films. The SEM images presented in these papers and the TEM image presented in this manuscript showed that all the materials have a uniform structure consisting of spherical nanoparticles. The nanoparticle size calculated from the SEM images is very close to the nanoparticle size obtained in this manuscript. Therefore, we concluded that there is no need to conduct TEM studies for 3TiO_2_–97SnO_2_ and 5TiO_2_–95SnO_2_ films, since the crystallite sizes of these materials have already been determined in the referenced papers based on the SEM measurements. Therefore, we can use the nanocrystallite size estimates presented in the referenced papers for the detailed analysis of the gas-sensing properties in this manuscript.

The distribution of titanium oxide particles in the film is uniform, and no large agglomerates were detected. When analyzing individual nanoparticles from HR TEM images (Figure 2c), interplane distances of 0.334 nm were found, which corresponds to the (110) cassiterite plane and is expected for a material consisting mainly of tin dioxide, as well as 0.32 nm, which corresponds to the (110) rutile TiO_2_ plane (red box in Figure 2c).

The EDX method confirmed the composition of the film: the distribution of the mass percentages of titanium, tin, and oxygen were 0.6, 44.2, and 55.2 at.%, respectively (Figure 2d–f), which correspond to the amounts of substances introduced.

The phase composition of thin TiO_2_-SnO_2_ films was studied by the X-ray diffraction method in our work [46]. According to the XRD data, the materials have a predominantly cassiterite structure, which is expected for materials containing a small amount of the introduced titanium dioxide additive [46]. No peaks related to the Ti-containing phase were detected, which can be explained by its highly dispersed state and small amount. When a small amount of titanium dioxide additive is introduced into SnO_2_, the particle size decreases sharply from 29 to 19 nm for 1TiO_2_–99 SnO_2_. With an increase in the concentration of titanium dioxide from 1 to 5%, an increase in particle size is observed. Thus, with the addition of a second oxide additive, the crystallite sizes of the main phase decrease compared to the pure phase. This can be explained: when a modified substance is introduced into the main phase, the contact crystallite surface of the main phase decreases, and the total free energy at the interphase boundaries decreases. This reduces the crystal growth rate.

**Figure 3:**
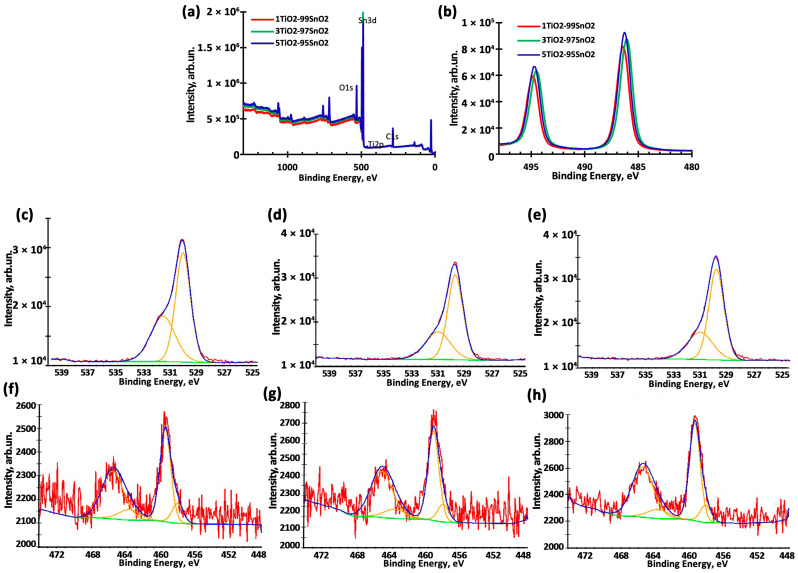
XPS spectra of TiO_2_–SnO_2_ films (**a**), high-resolution XPS spectrum Sn3d5 (**b**), O 1s (**c**–**e**), Ti 2p (**f**–**h**), for 1TiO_2_–99SnO_2_ (**c**,**f**), 3TiO_2_–97SnO_2_ (**d**,**g**), 5TiO_2_–95SnO_2_ (**e**,**h**). (**c**–**h**) Red line—raw data; green—background; orange—fitted components (explanations are given in the text); blue line—envelope.

Figure 3a presents the survey XPS spectra of TiO_2_–SnO_2_ films, showing the presence of C, O, Ti, and Sn elements. The quantitative distribution of elements in the samples’ surface layers with different atomic ratios was determined by fitting the high-resolution spectra using the Lorentzian–Gaussian model (Lorentzian function contribution—30%) and the Shirley background. Sensitivity factors were calculated based on the standard Scofield coefficients. Based on these data, asymmetric spectra of O1s and Ti2p were fitted to the ground levels. The Sn3d spectra were not adjusted, as they were symmetrical—Figure 3b. In addition, it was determined that the sample surface contains carbon atoms in approximately the same amount—about 40% of the total number of atoms. That is, the carbon content does not depend on the composition of the films, so the carbon spectra were not considered in detail.

Analysis of the Sn3d spectra showed that tin ions can be present in different oxidation states. Thus, for the 1TiO_2_–99SnO_2_ film, the binding energies of 486.58 and 494.98 ± 0.2 eV are characteristic of the Sn3d5/2 and Sn3d3/2 peaks, which is typical of tin oxide SnO_2_. This is also confirmed by the difference between these energy levels, equal to 8.4 ± 0.2 eV [47,48]. For the 5TiO_2_–95SnO_2_ film, this difference is also equal to 8.4 ± 0.2 eV. But for the 3TiO_2_–97SnO_2_ film, this difference is somewhat smaller and equal to 8.3 ± 0.2 eV, and the binding energies of the Sn3d5/2 and Sn3d3/2 peaks are 486.18 and 494.48 ± 0.2 eV, which may indicate the existence of tin atoms in the Sn^2+^ state [4,48].

The high-resolution spectra are shown in Figure 3c–h, and the parameters of the main peaks O1s and Ti2p, calculated as a result of fitting, are presented in Table 1. The values of χ2 (chi square) for the Ti2p and O1s XPS spectra due to the noise level of XPS spectra are 1.47 and 1.38, 1.93 and 1.44, and 1.48 and 1.23 for the films 1TiO_2_–99SnO_2_, 3TiO_2_–97SnO_2_, and 5TiO_2_–95SnO_2_, respectively. This indicates a more than 97% coincidence of the experimental XPS spectra with the curve obtained by deconvolution of the theoretical spectra. From the high-resolution spectra of the photoelectron line O1s (Figure 3c–e), it is evident that the main peak in the region of 530.2 ± 0.2 eV corresponds to the oxidized state of metal oxides. The second, less-intense peak in the region of 531.2 ± 0.2 eV corresponds to the oxygen vacancies in TiO_2_–SnO_2_ films [4,48].

The Ti2p photoelectron spectra (Figure 3f–h) showed that the energies of the main lines for the Ti2p1/2 and Ti2p3/2 peaks are close to 464.5 and 458.8 ± 0.2 eV, which corresponds to TiO_2_ [48]. At the same time, for all TiO_2_-SnO_2_ films, there are minor peaks with energies close to 457.28 and 462.82 ± 0.2 eV for the Ti2p1/2 and Ti2p3/2 peaks, respectively, which indicate the presence of TiO_x_ oxides [48]. The 3TiO_2_-97SnO_2_ film has the closest energies to these values (Figure 3g and Table 1). Thus, the analysis of the XPS spectra indicates that titanium oxides, TiO_x_, may exist in the TiO_2_-SnO_2_ films. The closest match in energy is observed for the 3TiO_2_-97SnO_2_ film, in which tin ions can be in the Sn^2+^ state. The existence of these levels can also lead to allowed states in the band gap of the semiconductor [47,48].

The distribution of metal ions in the samples, presented in Table 1, was also determined from the spectra. It is evident that with an increase in the titanium content in the films, the number of Ti^3+^ ions decreases.

**Figure 4:**
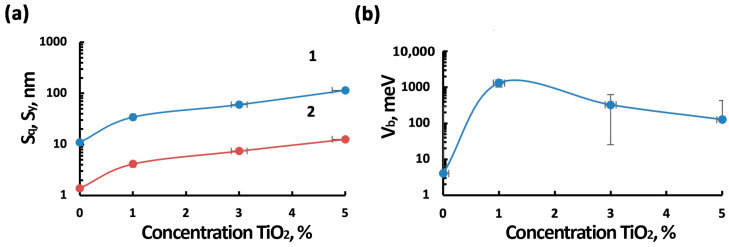
Dependence: (**a**) the roughness parameters S_q_ (curve 1) and S_y_ (curve 2); (**b**) the maximum values of the surface potential difference from the titanium ions concentration in the film.

Studies of the electrophysical and surface properties (AFM and KPFM studies) of TiO_2_–SnO_2_ nanocomposite films were carried out by us previously [44,49]. AFM studies (Figure 4) have shown that the films have a granular structure, with a peak-to-peak height difference of (S_y_) 11–114 nm. In general, when the titanium concentration in the film decreases, the roughness decreases from 114 to 11 nm. However, the surface of the 5TiO_2_–95SnO_2_ film has a higher roughness than the surface of the 3TiO_2_–97SnO_2_ and 1TiO_2_–99SnO_2_ films. KPFM studies have shown that the maximum values of the potential barrier (V_b_) are observed in 1TiO_2_–99SnO_2_ films, in which the maximum magnitude difference can reach 1325 meV, and the average value of the potential barrier (V_bmid_) is 141 meV. Lower (3.6–4 times and 8.3–10.5 times) values of potential barriers are observed in 3TiO_2_–97SnO_2_ films and 5TiO_2_–95SnO_2_ films, respectively. For comparison, Figure 4 shows the corresponding parameter values for the SnO_2_ film.

Electrophysical measurements have shown [44,49] that as the temperature increases, the resistance of TiO_2_–SnO_2_ film samples decreases, which indicates a semiconductor conduction mechanism. The activation energy of conductivity (E_a_) corresponds to half of the ionization energy of the impurity (defective) level and was calculated using the Arrhenius equation for the temperature ranges shown in Table 2. Calculations of the activation energy of conductivity showed that in the temperature range from RT to 120 °C, the E_a_ values are the same for all films (0.33 eV). In other temperature ranges, the 1TiO_2_–99SnO_2_ film has lower E_a_ values, and with an increase in the TiO_2_ content in the films, the activation energy values increase.

Since in SnO_2_ the energy level formed by oxygen vacancies lies 0.14–0.15 eV below the bottom of the conduction band [50], and in the band gap of TiO_2_, the allowed energy levels with different energy values below the bottom of the conduction band are created by the oxygen vacancies of Ti^3+^ and Ti^2+^ [51]; in our case, the calculated values E_a_ can be associated with the oxygen vacancies of Ti^3+^ and Ti^2+^.

**Figure 5:**
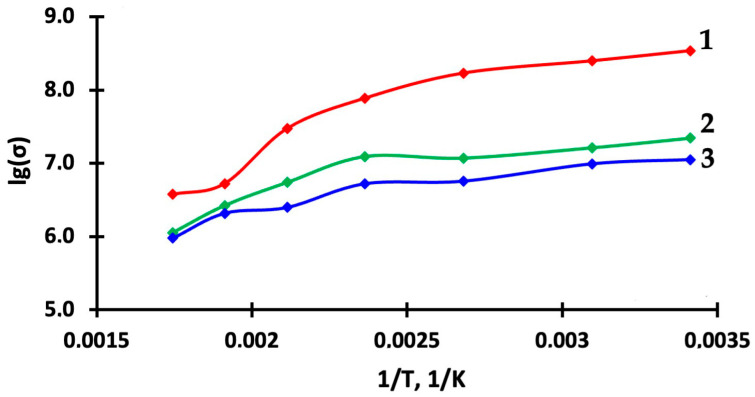
Dependence of the conductivity logarithm of 1TiO_2_–99SnO_2_ (1), 3TiO_2_–97SnO_2_ (2), and 5TiO_2_–95SnO_2_ (3) films on the reverse temperature.

Measurements of the sensor structure conductivity from temperature when exposed to light activation are shown in Figure 5. Calculations of the conduction activation energy were performed, which showed lower values of the conduction activation energy (0.05–0.12 eV) at low operating temperatures of 20–150 °C for 1TiO_2_–99SnO_2_, 3TiO_2_–97SnO_2_, and 5TiO_2_–95SnO_2_ films over the entire temperature range—Table 2. However, for 1TiO_2_–99SnO_2_, 3TiO_2_–97SnO_2_, and 5TiO_2_–95SnO_2_ films at temperatures of 150–300 °C, the E_a_ values become higher (0.42, 0.33, and 0.24 eV, respectively). This fact indicates that at low operating temperatures for 1TiO_2_–99SnO_2_ and 3TiO_2_–97SnO_2_ films, the effect of light activation due to charge carrier photogeneration negates the effect of charge carrier thermal generation. In this temperature range, a low-temperature drift of sensor parameters can be expected.

### 3.2. Optical Properties

**Figure 6:**
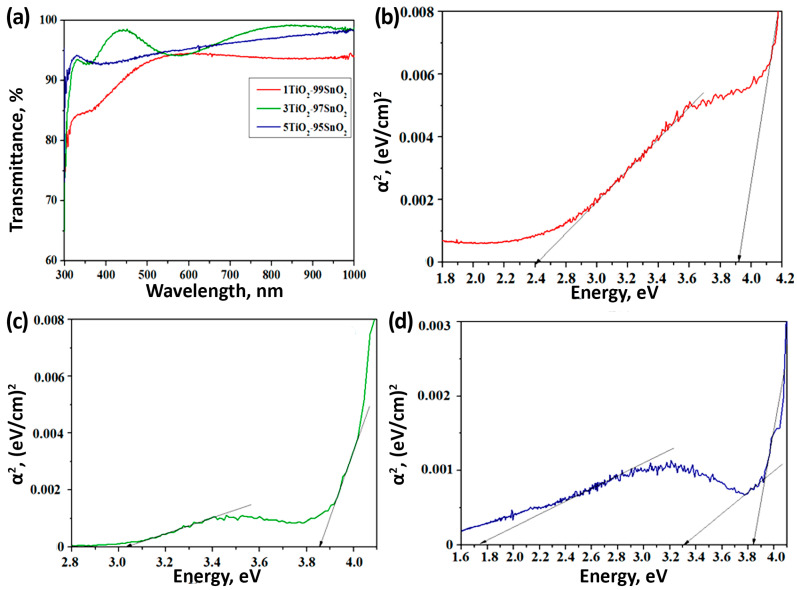
Optical transmission spectra (**a**) and band gap estimation for 1TiO_2_–99SnO_2_ (**b**), 3TiO_2_–97SnO_2_ (**c**), and 5TiO_2_–95SnO_2_ thin films (**d**).

Evaluation of the optical properties (Figure 6a) of the synthesized TiO_2_–SnO_2_ thin films showed that all of the materials are optically transparent in the visible light range (the transmission coefficient is no lower than 80%). The maximum transparency coefficient (99%) is observed for the 3TiO_2_–97SnO_2_ material at 436 nm; for the 1TiO_2_–99SnO_2_ material, transparency is shown at 90% in the wavelength range of 500–1000 nm. In the visible range, the values of the transmission coefficient for all materials are close, which allows us to conclude that the additive has a positive effect on the optical transparency of the synthesized film nanomaterials.

The maximum E_g_ value for the 3TiO_2_–97SnO_2_ film will be 3.83 eV (black arrow in the figure)—Figure 6c. However, the presence of an Urbach tail indicates that the small crystallite size leads to a blurring of the band gap [52]. A similar situation is also observed for 3TiO_2_–97SnO_2_ and 5TiO_2_–95SnO_2_ films. It can be noted that the Tauc plots indicate the existence of other allowed states in the band gap of TiO_2_–SnO_2_ films in the range of 1.8–3.1 eV (shown by tangent lines), which may also be related to the structural disorder caused by the boundaries of nanograins and the presence of other structural defects [53]. It is known that in structures with an oxygen deficiency of SnO_2−x_, the band gap can decrease from 3.2 eV to 2.16 eV, depending on the ratio of Sn^4+^/Sn^2+^ oxygen vacancies [47]. It was shown in [48] that in TiO_2_–SnO_2_ heterostructures, the effective band gap can decrease since the Ti^3+^ states in TiO_2_ create energy levels below the bottom of the conduction band, and the Sn^2+^ states create energy levels 0.7–1.4 eV above the top of the valence band.

### 3.3. NO_2_ Gas-Sensing Performance of TiO_2_-SnO_2_-Based Gas Sensors

**Figure 7:**
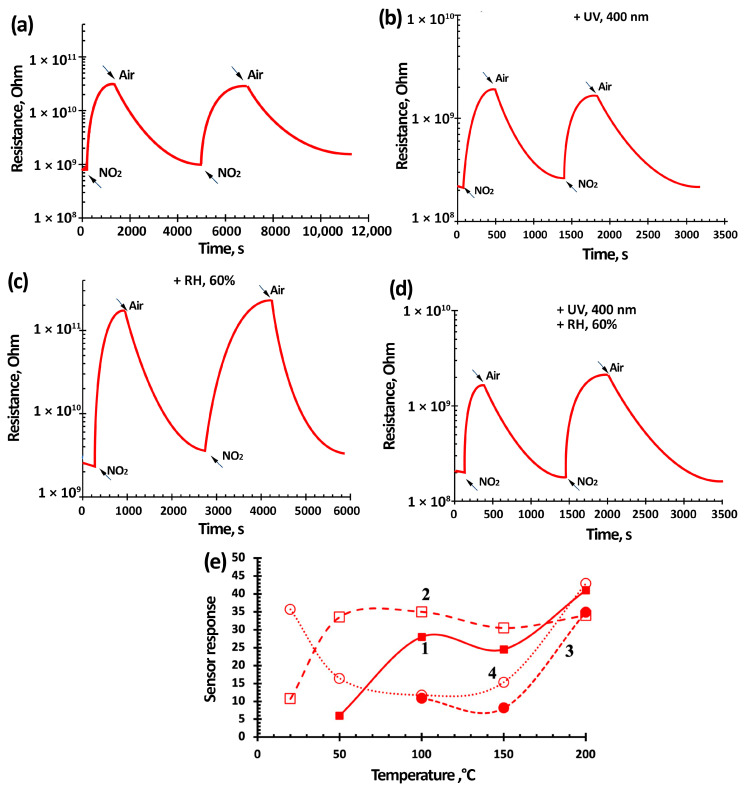
Kinetics of the resistance change (**a**–**d**) and temperature-dependence of the sensor response (**e**) based on the 1TiO_2_–99SnO_2_ film at a temperature of 100 °C and exposure to 7.7 ppm NO_2_ (curve 1) and under light activation ((**b**,**d**) and (**e**), curves 2 and 4), under the influence of 60% RH ((**c**,**d**) and (**e**), curves 3 and 4).

**Figure 8:**
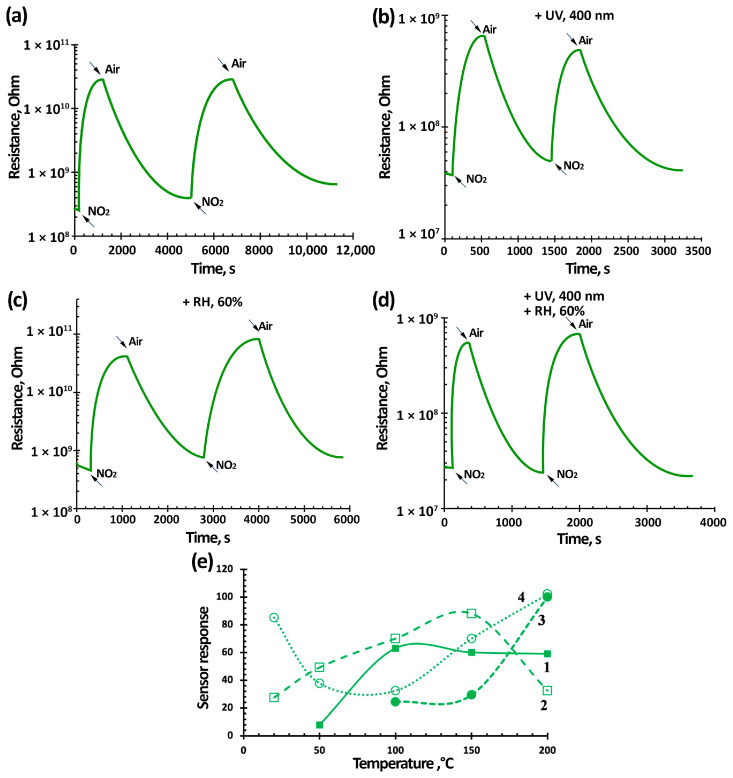
Kinetics of the resistance change (**a**–**d**) and temperature-dependence of the sensor response (**e**) based on the 3TiO_2_–97SnO_2_ film at a temperature of 100 °C and exposure to 7.7 ppm NO_2_ (curve 1) and under light activation ((**b**,**d**) and (**e**), curves 2 and 4), under the influence of 60% RH ((**c**,**d**) and (**e**), curves 3 and 4).

**Figure 9:**
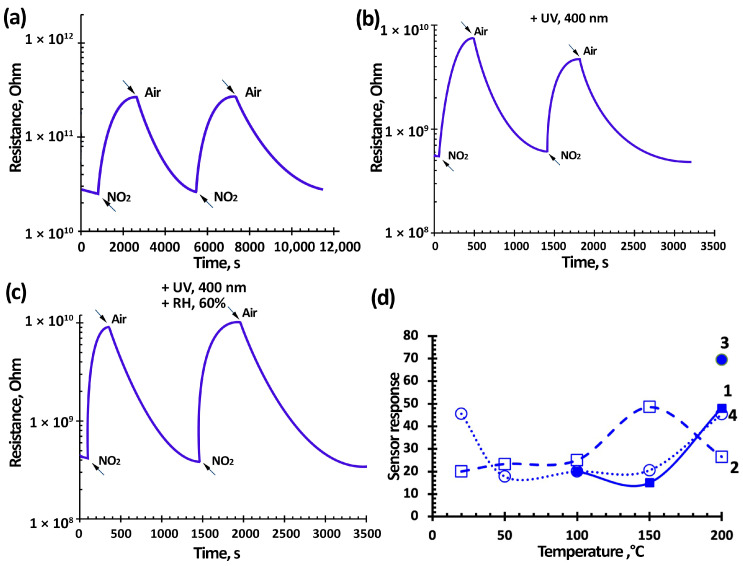
Kinetics of the resistance change (**a**–**c**) and temperature-dependence of the sensor response (**d**) based on the 5TiO_2_–95SnO_2_ film at a temperature of 100 °C and exposure to 7.7 ppm NO_2_ (curve 1) and under light activation ((**b**,**c**) and (**d**), curves 2 and 4), under the influence of 60% RH ((**c**) and (**d**), curves 3 and 4).

The gas-sensitivity determination of sensors based on TiO_2_–SnO_2_ films for four gases—nitrogen dioxide (7.7 ppm), ethanol vapor (100 ppm), carbon(II) monoxide (500 ppm), and methane (1000 ppm)—at 200 °C was previously performed. The results showed that the maximum gas-sensitivity values were for NO_2_ (Figure 7e, Figure 8e and Figure 9d). The response to C_2_H_5_OH and CO was about 1–2, and there was no response to CH_4_. In this regard, it was decided to perform the tests with NO_2_ in the temperature range from room temperature to 200 °C. The gases selected for analysis are the most common pollutants that are controlled in manufacturing plants. These include inorganic gases such as NO_2_ and CO and organic gases like C_2_H_5_OH and CH_4_. Additionally, a variety of gases with different chemical properties have been selected, including oxidizing gases such as NO_2_, and reducing gases like CO and CH_4_.

Figure 7, Figure 8 and Figure 9 show typical responses of sensors based on TiO_2_–SnO_2_ films to NO_2_ at a concentration of 7.7 ppm (without and with exposure to 60% humidity) and when exposed to light activation with a wavelength of 400 nm (without and with exposure to 60% humidity) at an operating temperature of 100 °C. Figure 7e also shows the temperature-dependences of the response (S).

Figure 7, Figure 8 and Figure 9 show that the TiO_2_–SnO_2_ sensors’ response to 7.7 ppm of NO_2_ exposure at an operating temperature of 200 °C is 20–30% higher than the response of sensors when exposed to light activation. As can be seen from Figure 4 and Table 2, at this temperature, the effect of light activation leading to charge carrier photogeneration is still insufficient. However, already at temperatures below 150 °C, the contribution of photogenerated charge carriers becomes noticeable, which leads to an excess of the sensor response when exposed to light activation over the sensor responses without this effect for all of the sensors under study. It is known that the affinity of the NO_2_ molecule to an electron is significantly higher (2.27 eV) than that of the O_2_ molecule (0.45 eV). In this regard, possible processes are the adsorption of NO_2_ molecules with subsequent ionization and the displacement of some chemisorbed oxygen forms from the semiconductor oxide surface [6]:

(5) NO_2_(gas) → NO_2_(ads) + e^−^ → NO_2_^−^(ads)

(6) NO_2_(gas) + O_2_^−^(ads) → NO_2_^−^(ads) + O_2_(gas),

In our case, due to the effect of light activation with a wavelength of 400 nm, this process has shifted towards lower temperatures.

In addition, when exposed to light activation, the operating temperatures of sensors decrease down to RT (20 °C)—Figure 7e, Figure 8e and Figure 9d. At low operating temperatures, the nanocomposite structure of TiO_2_–SnO_2_ films, which consist of TiO_2_ and SnO_2_ nanocrystallites and form n-n heterojunctions, begins to play a significant role (Figure 2). There is a considerable potential barrier at the heterojunction boundary, which reaches 1325 meV for the 1TiO_2_–99SnO_2_ film and 326 meV for the 3TiO_2_–97SnO_2_ film. The latter means that there is a strong electric field near and at the heterojunction boundary, which promotes the course of surface chemisorption processes. A strong surface electric field contributes to an increase in the size of the Urbach tail at the fundamental absorption edge of the studied material, as can be seen in Figure 9.

The influence of humidity, as is known, leads to a significant decrease in the response value, which is clearly seen in the temperature-dependences of the response value at 60% RH (Figure 7e, Figure 8e and Figure 9d). This is due to the adsorption of water molecules on the gas-sensitive material surface and their desorption with the formation of OH_ads_ hydroxogroups [29,36,37]. Water molecules and OH_ads_ hydroxogroups occupy surface adsorption centers, preventing the adsorption of NO_2_ molecules. The effect of 60% RH leads to the absence response from sensors based on 1TiO_2_–99SnO_2_ and 5TiO_2_–95SnO_2_ films; there is no response even at temperatures of 150 °C and below.

However, light activation acting simultaneously with humidity in the temperature range of 50–150 °C leads to the sensors’ response value being 1.01–1.88-times higher than the sensors’ responses operating only in a humid environment. Apparently, the effect of light activation at 60% RH promotes not only the desorption of O_2(ads)_ molecules and the release of adsorption centers, but also the desorption of water molecules and OH_ads_ hydroxogroups.

**Figure 10:**
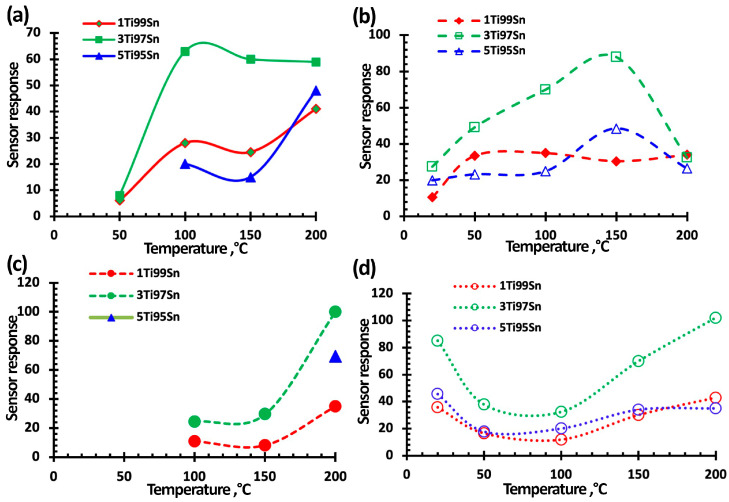
Temperature-dependence of the sensors’ response for 1TiO_2_–99SnO_2_, 3TiO_2_–97SnO_2_, and 5TiO_2_–95SnO_2_ to an exposure of 7.7 ppm NO_2_: (**a**) without influence; (**b**) under light activation; (**c**) at 60% RH, (**d**) at 60% RH and under light activation. The operating temperature is 100 °C.

Figure 10 shows that the responses of sensors based on TiO_2_–SnO_2_ films increase when exposed to humidity and light activation both at temperatures below 50 °C and at temperatures above 200 °C. It is known that at temperatures from 200 °C and above, water molecules and OH groups are actively desorbed [54], which leads to a higher response. This is confirmed by the close response values at a temperature of 200 °C with and without exposure to light activation. This means that the influence of humidity is minimized and surface reactions proceed according to Equations (5) and (6).

At RT, the response of a sensor operating at 60% humidity and activated by light increases by 2.17–2.46 times compared with the response at 50 °C. According to [55,56,57], water molecules are present on the MOS surface at temperatures below 100–150 °C. Quantum chemical calculations have shown that the process of dissociation of an isolated H_2_O molecule is energetically advantageous [58]. Upon dissociation, a proton and a hydroxogroup are formed. The hydroxogroup interacts with the lattice tin atom (Sn_lat_), and the proton interacts with the lattice oxygen (O_lat_) to release an electron:

(7) $$H_{2}O+{\mathrm{Sn}}_{\mathrm{lat}}+O_{\mathrm{lat}}\;{↔Sn}_{lat}^{\delta +}-{OH}^{\delta -}+(O_{\mathrm{lat}}-H)+ē$$

At temperatures below 50 °C, a strong electric field begins to take effect, which occurs at the n-n-heterojunction boundary due to the high values of potential barriers. Thermodynamic calculations [59] also showed the possibility of OH group separation in the presence of a strong surface electric field. In [13], a calculation was performed and it was shown that the energy of interaction between the NO_2_ molecule and the charged adsorption center is sufficient for the ionization of NO_2_ molecules to occur, followed by dissociation and/or the appearance of atomic oxygen ions [6]:

(8) 2 NO_2_(gas) → NO_2_(ads) + e^−^ → 2 NO(gas) + O_2_^−^(ads)

(9) 2 NO_2_(gas) + O_2_^−^(ads) + 2e^−^ → NO_2_^−^(ads)+ 2 O^−^(ads).

The electron released as a result of reaction (7) can be involved in reactions (8) and (9). Thus, these reactions lead to high response values.

Thus, the mechanism of NO_2_ molecules’ interaction without and under light activation (400nm, <0.2 mW/cm^2^) or humidity (60% RH), as well as their combined action on TiO_2_–SnO_2_ films, can be as follows. It is known that TiO_2_ has a band gap (E_g_TiO_2_), a work function (φTiO_2_), and an electron affinity (χTiO_2_) equal to 3.2, 5.1, and 4.21 eV, respectively. For SnO_2_, these values (E_g_SnO_2_, φSnO_2_, and χSnO_2_) are 3.6, 4.7, and 4.5 eV, respectively [21,60]. During the formation of a heterojunction between TiO_2_ and SnO_2_ nanocrystallites, electrons pass from SnO_2_ to TiO_2_. Since TiO_2_ crystallites are surrounded by SnO_2_ crystallites (Figure 2), as a result, TiO_2_ crystallites may be in a degenerate state, as we observed during the formation of the ZnO–SnO_2_ heterojunction [13]. In this case, high surface potentials are formed at the n-n-heterojunction (Figure 4b). It is also known that the increased oxygen content on the surface of TiO_2_ leads to an increase in the work function to φTiO_2_ = 5.35 eV. For the hydroxylated TiO_2_ surface, in which the vacancies are filled with the OH hydroxogroup, the work function is φTiO_2_ = 4.9 eV [61]. In any case, φTiO_2_ > φSnO_2_, which means that the mechanism of the surface potential formation will not change. According to Equations (5)–(7), the adsorbed NO_2_ molecule captures electrons from the conduction band of SnO_2_ and TiO_2_, which leads to a decrease in the near-surface electron concentration. The resistance of the heterojunction increases in this case, as can be seen in the dynamics of the response of the gas sensors.

The figures also show that when exposed to light activation, the performance of sensors based on TiO_2_–SnO_2_ films improves in some cases, which confirms the conclusions [62]. Thus, for a sensor based on a 1TiO_2_–99SnO_2_ film, the response time t_0_._9_ improves by 10–15% at operating temperatures below 200 °C. For a sensor based on a 3TiO_2_–97SnO_2_ film, the response time t_0.9_ at operating temperatures below 200 °C improves by 20–40% and is in the range of 90–180 °C.

Figure 10 shows that the sensor based on the 3TiO_2_–97SnO_2_ film has the maximum response values. At operating temperatures of 100–200 °C, the response value is 1.4–2.3-times higher than the response of sensors based on other films. At a temperature of 50 °C, the response values of sensors based on 1TiO_2_–99SnO_2_ and 3TiO_2_–97SnO_2_ films become close—Figure 10a.

The sensors’ response time (recovery) at an operating temperature of 100 °C and concentrations 7.7 ppm, regardless of the film composition, was 914 ± 10 s (3086 ± 10 s), when exposed to radiation it decreased to 344 ± 20 s (888 ± 20 s), at 60% RH it was 741 ± 6 s (1371 ± 100 s), and at 60% RH, the light activation decreased to 351 ± 20 s (1048 ± 20 s). It can be seen that the light activation effect led to a 2.6-fold decrease in response time, and a 2.1-fold decrease at 60% RH. The recovery time improved 3.4 times, and at 60% RH it was 1.8 times. This can be explained by the fact that the adsorbed water molecules were less actively desorbed when exposed to light activation compared to oxygen molecules. As expected, the response/recovery time decreased to 225 ± 10 s (1160 ± 10 s) when the NO_2_ concentration was reduced to 0.77 ppm, while it decreased to 145 ± 20 s (520 ± 20 s) when exposed to radiation, and decreased to 132 ± 20 s (448 ± 20 s) at 60% relative humidity and light activation. At room temperature, the response/recovery times for a NO_2_ concentration of 0.77 ppm, exposed to radiation, were 225 ± 20 s (440 ± 20 s); and for 0.2 ppm NO_2_, the response/recovery times were 144 ± 20 s (180 ± 20 s). In general, the response/recovery times decrease as the NO_2_ concentration decreases. This is explained by the low competition of gas molecules during their adsorption/desorption on the surface of the gas-sensitive material.

**Figure 11:**
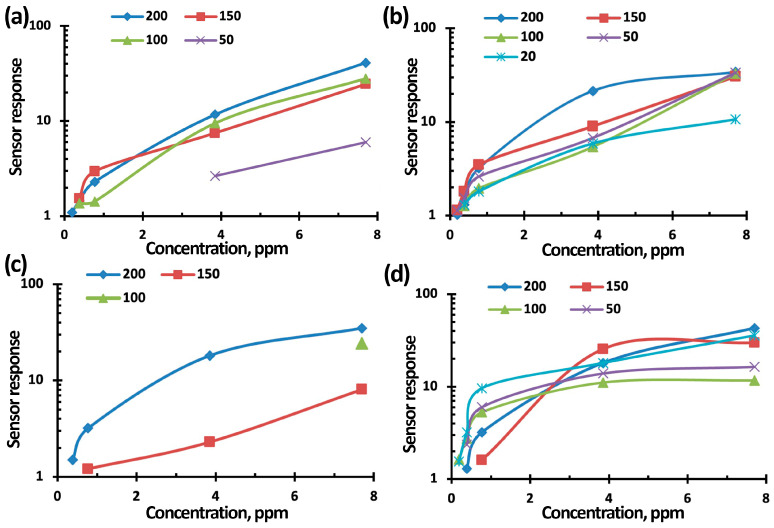
Concentration-dependence of the 1TiO_2_–99SnO_2_ sensor response to NO_2_ without exposure (**a**) and when exposed to light activation (**b**,**d**); at 60% RH (**c**,**d**).

**Figure 12:**
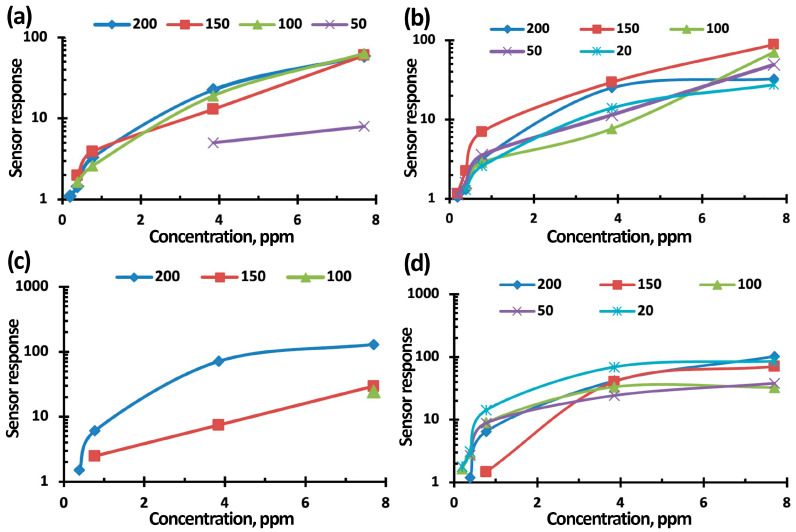
Concentration-dependence of the 3TiO_2_–97SnO_2_ sensor response to NO_2_ without exposure (**a**) and when exposed to light activation (**b**,**d**); at 60% RH (**c**,**d**).

**Figure 13:**
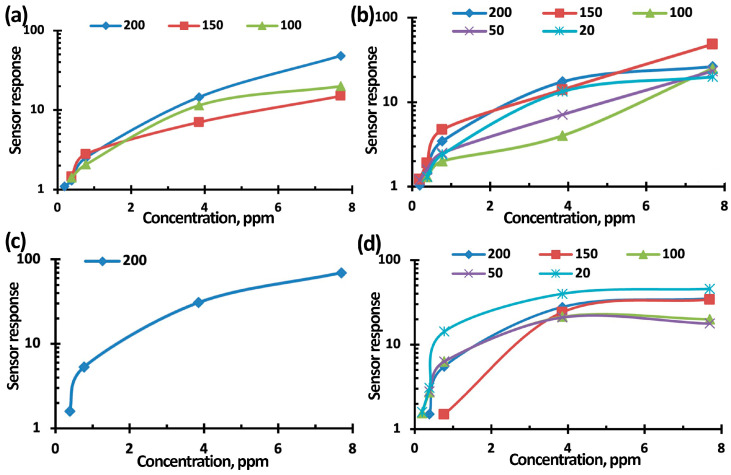
Concentration-dependence of the 5TiO_2_–95SnO_2_ sensor response to NO_2_ without exposure (**a**) and when exposed to light activation (**b**,**d**); at 60% RH (**c**,**d**).

Figure 11, Figure 12 and Figure 13 show the concentration-dependences of the sensors’ responses based on TiO_2_–SnO_2_ films exposed to NO_2_ with concentrations of 0.2–7.7 ppm when exposed to light activation with a wavelength of 400 nm, 60% RH, and an operating temperature of 100 °C. The measurement error was no more than 5%. It can be seen from Figure 11, Figure 12 and Figure 13 that the sensor based on the 3TiO_2_–97SnO_2_ film has the maximum response. The lower limit of sensitivity can be considered a concentration of 0.2 ppm.

In our work, it was determined that all of the concentration curves are well aligned in bilogarithmic coordinates lnS–lnC. It follows from this that the adsorption of NO_2_ molecules under any conditions obeys the Freundlich isotherm [29]. The experimental parameters of the Freundlich equation for all curves are presented in Table S1. The theory of adsorption for this case says that the energy distribution of active adsorption centers obeys an exponential law. It can also be concluded that exposure to light activation and the presence of humidity, as well as their simultaneous effects, do not affect the adsorbent, but can only change the number of adsorption centers.

The study was conducted using sensors manufactured in five different lots. The measurement results showed that the sensor responses for the same gas-sensitive material film compositions, but from different lots, were reproducible within 10–15%.

**Figure 14:**
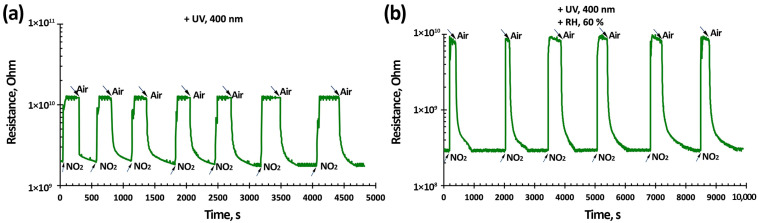
3TiO_2_–97SnO_2_ sensors’ temporal stability when exposed to 3.85 ppm NO_2_ (RT) under light activation (**a**) and under light activation and 60% RH (**b**) on different days.

Figure 14 shows graphs of the 3TiO_2_–97SnO_2_ sensors’ temporal stability during measurements of NO_2_ with a concentration of 3.85 ppm at RT and light activation (Figure 14a) and under light activation and 60% RH (Figure 14b). The drift of sensor response during the study time is not noticeable. There was a spread of meanings via seven responses of no more than 5%. Similar measurements were repeated several times over the course of a month and the results were confirmed (Figure S1).

Temporal stability studies were conducted on a stand with a smaller camera volume, which contained sensors. The sensor response time for RT and light activation was 162 s, and the recovery time was 350 s. Under light activation and 60% RH at RT, the sensor response/recovery time was 245/460 s.

Table 3 shows a comparison of the characteristics of sensors based on SnO_2_. Interestingly, there are few studies devoted to the synthesis of materials with gas-sensitive properties at RT.

At the same time, judging by the citations, interest in this topic is quite high. From Table 3, it can be seen that under the influence of humidity, the sensors show good responses but at temperatures above 100 °C. Thus, a sensor based on bi-layer SnO_2_/TiO_2_ n-n heterostructures showed a response of 881 to exposure to 12 ppm NO_2_ at 150 °C and 50% RH [70], and a sensor based on SnO_2-x_ nanocrystals at 100 °C and 30% RH showed a response of 70 to 0.5 ppm [64]. The authors of [43] obtained the best detection limit equal to 4 ppb NO_2_ with a low sensitivity of 0.6%. At a concentration of 0.5 ppm, the sensitivity of the sensor is only 21.9%. In addition, its design, as we wrote above, may not be durable. These results are the closest to those obtained in this work for a sensor based on a 3TiO_2_–97SnO_2_ film: when exposed to 7.7 ppm of NO_2_ at RT, activation by light, and 60% RH, the response is 85. The sensitivity at a concentration of 0.2 ppm is 1.8, which is more than 3.7-times higher than that of the sensor described in [43], at a NO_2_ concentration of 500 ppb. And compared to the response of another sensor operating at room temperature described in [63], the response of our sensor at the same concentration is 8.5-times better. As can be seen, due to the formation of a composite TiO_2_–SnO_2_ film structure using the oxidative pyrolysis method, it is possible to reduce the operating temperature to RT and increase the response by 3.7–8.5 times compared to the currently known result. It should also be noted that the synthesis method (oxidative pyrolysis) is simpler than hydrothermal or sol–gel synthesis and provides good reproducibility of material properties. Also in this work, the sensitivity threshold of the sensor has been lowered to 0.2 ppm relative to NO_2_ with a response of 1.8.

The sensor based on the 3TiO_2_–97SnO_2_ film has the best characteristics. This can be explained by several reasons. First of all, the surface potential of the 3TiO_2_–97SnO_2_ films V_b_ is one of the highest (Figure 4b). Secondly, the activation energy of conductivity E_a_ at temperatures closer to room temperature is the smallest (Table 2). Thirdly, the energy of LED radiation at the main wavelength of 400 nm is 3.1 eV. This corresponds to one of the allowed levels in the band gap of the semiconductor (about 3.1 eV). Apparently, light quanta with the specified wavelength are absorbed at the allowed energy levels and excite electrons, which can jump to the conduction band due to the low activation energy and surface electric field. When NO_2_ molecules are adsorbed, these electrons are more actively captured by the molecules and change the conductivity of 3TiO_2_–97SnO_2_ films more strongly than in films with other compositions.

Thus, the sensors developed in this work have peak characteristics when detecting low concentrations of NO_2_ at RT and 60% RH. This makes them promising for the determination of NO_2_ in the air of populated areas, in the work areas of industrial enterprises, and in the non-invasive diagnosis of various human diseases.

## 4. Conclusions

TiO_2_–SnO_2_ thin films were synthesized by oxidative pyrolysis and formed using nanoparticles with a size from 19 nm (1TiO_2_–99SnO_2_) to 29 nm (5TiO_2_–95SnO_2_). The films’ composite structure was confirmed by the HR TEM method, and particles with interplane distances characteristic of TiO_2_ and SnO_2_ were detected. The quantitative composition of the materials was confirmed by the XPS and EDX methods. The obtained films are optically transparent, and the maximum band gap is characteristic for 3TiO_2_–97SnO_2_ films and is 3.83 eV. Moreover, all composite films are characterized by the presence of Urbach tails, which explains the presence of additional energy transitions in the band gap. The Urbach tail also explains the existence of a strong surface electric field formed by large (up to 1325 meV for 1TiO_2_–99SnO_2_ and 326 meV for 3TiO_2_–97SnO_2_) surface potentials in the region of TiO_2_–SnO_2_ heterojunctions detected by the KPFM method.

When studying the gas-sensitive properties, it was revealed that sensors based on 3TiO_2_–97SnO_2_ films have maximum responses in all of the studied modes. For sensors based on 1TiO_2_–99SnO_2_ and 3TiO_2_–97SnO_2_ films, the effect of low-intensity radiation (400 nm, intensity < 0.2 mW/cm^2^) leads to sensitivity to NO_2_ at RT and an increase in the sensor response by 1.2–5 times at operating temperatures of 50–150 °C. The effect of 60% RH significantly impairs the response value, sensitivity limit, and operating temperature of all sensors. The combined effect of radiation and 60% RH was found, leading to a sharp increase in the response at RT by 2.2–4.5 times compared with the response of sensors under only light activation. The explanation of this effect is related to the processes of dissociative adsorption of water molecules and ionization of hydroxogroups, which lead to an improvement in the interaction of NO_2_ molecules on the surface of the TiO_2_–SnO_2_ film. The sensor based on 3TiO_2_–97SnO_2_ films has the best characteristics.

The maximum response for RT, light activation, and 60% RH at 0.2 ppm NO_2_ is 1.8, and for exposure to 7.7 ppm NO_2_ it is 85. The lower sensitivity limit is 0.2 ppm NO_2_.

Thus, the TiO_2_–SnO_2_ composite films obtained in the work have a high sensitivity in photoactive gas sensors operating at RT and providing the detection of NO_2_ with low concentrations at 60% RH. This makes them promising for monitoring the air quality of populated areas, for industrial enterprises, and in the non-invasive diagnosis of human diseases.

## Figures and Tables

**Table 1:** Characteristics of XPS spectra and concentration of titanium and tin atoms/ions in the TiO_2_-SnO_2_ films.

Sample	O1s		Sn 3d		Ti2p3 (Ti^3+^)		Ti2p3 (Ti^4+^)		Concentration of Atoms/Ions			
	O_v_	Oxide	3d3\2	3d5\2	2p3/2	2p1/2	2p3/2	2p1/2	Sn	Ti	Ti^3+^	Ti^4+^
1TiO_2_–99SnO_2_	531.78	530.28	494.98	486.58	457.48	462.88	457.78	464.78	98.17	1.83	17.7	82.3
3TiO_2_–97SnO_2_	531.20	530.08	494.48	486.18	457.38	462.88	458.48	464.28	96.44	3.56	16.5	83.5
5TiO_2_–95SnO_2_	531.38	530.18	494.68	486.28	457.38	462.88	458.58	464.58	95.28	4.72	15.0	85.0

**Table 2:** Conduction activation energies of TiO_2_–SnO_2_ films.

Thin Film	E_a_, eV			E_a_, eV (with Light Activation)	
	30–120 °C	120–230 °C	230–300 °C	20–150 °C	150–300 °C
1TiO_2_–99SnO_2_	0.33	0.56	0.51	0.12	0.42
3TiO_2_–97SnO_2_	0.33	0.68	0.57	0.05	0.33
5TiO_2_–95SnO_2_	0.33	0.76	0.63	0.06	0.24

**Table 3:** Comparison of the TiO_2_–SnO_2_ sensors characteristics.

Materials	Synthesis Method	Sensitivity or Response (NO_2_ Concentration)	Response/Recovery Time, s	Measurement Conditions		References
				Light Source	Temperature, Humidity	
SnO_2_ mono-layer array, particle size 20 nm	Hydrothermal	10 (10 ppm)	-	365 nm	RT 62% RH	[63]
SnO_2_ nanoparticles	Rheotaxial growth and its thermal oxidation	20% (1 ppm)	900/240	365 nm	RT, 30% RH	[64]
SnO_2−x_ nanocrystals, grain size 10 nm	Hydrothermal	70 (500 ppb)	230/88	-	100 °C, 26% RH	[65]
SnO_2_/ZnO heterostructure, ZnO nanowires 30–50 nm diameter, SnO_2_ rootstock 100–110 nm diameter	Thermal evaporation technique	390 (1 ppm)	-	-	30 °C	[66]
Sn–doped TIO_2,_ nanoparticle size 8 nm	Successive Ionic Layer Adsorption and Reaction Method	12% (10 ppm)	-	-	RT	[67]
0.05Pd/SnO_2_	Chemical method and ultrasonic exposure	3000 (5 ppm)	168/108	365 nm		[68]
WO_3_–SnO_2_ nanocomposites	Hydrothermal method	NO_2_, 10 ppm, 1167	39.51/98.07	-	150 °C	[69]
Bi-layers SnO_2_/TiO_2_ n-n heterostructures	Magnetron sputtering/L–B technique	NO_2_, 0.2 ppm, 847	26/58	-	123 °C	[70]
		NO_2_, 12 ppm, 881	-		150 °C, 50% RH	
Nanoporous two-layer TiO_2_/SnO_2_	two-step sol–gel process	NO, 4 ppb, 0.6%	35/85 s	365 nm, 3 μW/cm^2^	RT, 30% RH	[43]
		NO_2_, 500 ppb, 21.9%	100 /320 s			
3TiO_2_–97SnO_2_	Oxidative pyrolysis	NO_2_, 7.7 ppm, 85	245/460 s	400 nm 0.2 mW/cm^2^	RT, 60% RH	This work
		NO_2_, 0.2 ppm, 1.8	144/180 s			

## Data Availability

The data presented in this study are available upon request from the corresponding author.

## Supplementary Materials

The following supporting information can be downloaded at https://www.mdpi.com/article/10.3390/nano15110871/s1. Table S1. The values of the coefficients of the Freundlich equation. Figure S1. 3TiO_2_–97SnO_2_ sensors temporal stability (one month after the first measurement) when exposed to 3.85 ppm NO_2_ (RT) under light activation (a) and under light activation and 60% RH (b) in different days.
