# Supplementary material for: Excellent Room-Temperature NO2 Gas-Sensing Properties of TiO2-SnO2 Composite Thin Films Under Light Activation
Source: Nanomaterials (Basel). 2025 Jun 5;15(11):871. doi: 10.3390/nano15110871 (PMC12158011; doi:10.3390/nano15110871)
Supplement: Supplementary file 1 [file nanomaterials-15-00871-s001.zip › nanomaterials-3610602-supplementary.pdf]

Excellent room temperature NO_2 gas sensing properties of TiO_2-SnO_2 composite thin films under light activation

Victor V. Petrov [1*], Aleksandra P. Starnikova [1], Maria G. Volkova [2], Soslan A. Khubezhov [3], Ilya V. Pankov [4], Ekaterina M. Bayan [2]

[1] Southern Federal University, Institute of Nanotechnologies, Electronics, and Equipment Engineering, 347928 Taganrog, Russia; vvpetrov@sfedu.ru (V.V..P.); a.starnikova@mail.ru (A.P.S.)

[2] Southern Federal University, Department of Chemistry, 344090 Rostov-on-Don, Russia; ekbayan@sfedu.ru (E.M.B.); mvol@sfedu.ru (M.G.V.)

[3] Qingdao Innovation and Development Center, Harbin Engineering University, Shandong, Qingdao, 266000, China; soslan.khubezhov@gmail.com (S.A.Kh.)

[4] Southern Federal University, Institute of Physical and Organic Chemistry, Stachki Av. 194/2 Rostov-on-Don, Russia ipankov@sfedu.ru (I.V.P.)

* Correspondence: vvpetrov@sfedu.ru (V.V.P.)

The Freundlich equation used to extrapolate the measurement results shown in Fig. 11-13 has the form

$$S = k \cdot C^m, \qquad \text{(S1)}$$

где S – sensor response; C – concentration NO_2, ppm; k, m – Freundlich equation constants determined from experimental data.

Table S1 presents the values of the constants k and m of equation S1 for all curves in Fig. 11–13 that had more than two experimental points. Table S1 also presents the confidence level R2 of the extrapolation curve.

Table S1. The values of the coefficients of the Freundlich equation

1TiO_2–99SnO_2 sensors															
Exposure	Gas			Gas + light					Gas + 60%RH		Gas + light +60%RH				
T, °C	100	150	200	20	50	100	150	200	150	200	20	50	100	150	200
k	2.71	3.40	4.03	2.37	3.68	2.09	4.19	4.49	1.29	4.18	7.27	5.38	4.72	2.61	4.04
m	1.05	0.84	0.97	0.71	0.84	0.52	0.83	1.03	0.75	1.06	0.80	0.62	0.54	1.35	1.15
R^2	0.95	0.96	0.96	0.99	0.95	0.95	0.97	0.98	0.95	0.99	0.95	0.95	0.95	0.95	1.0
3TiO_2–97SnO_2 sensors															
k	4.35	4.98	5.24	3.44	4.66	3.27	7.67	4.60	2.86	7.57	12.14	7.75	7.85	2.60	6.28
m	1.22	1.05	1.12	1.02	0.97	0.79	1.14	1.03	1.01	1.50	1.11	0.83	0.86	1.74	1.41
R^2	0.98	0.95	0.98	1.0	0.96	0.98	0.99	0.98	0.95	0.98	0.96	0.95	0.95	0.97	0.98
5TiO_2–95SnO_2 sensors															
k	3.08	3.06	4.29	3.21	3.51	2.51	5.39	4.31	---	5.98	9.57	6.33	6.02	2.42	5.36
m	0.93	0.73	1.04	0.95	0.77	0.86	0.95	0.93	---	1.22	0.93	0.65	0.73	1.42	1.04
R^2	0.99	0.99	0.97	0.99	0.95	0.95	0.98	0.99	---	0.99	0.95	0.95	0.95	0.96	0.96

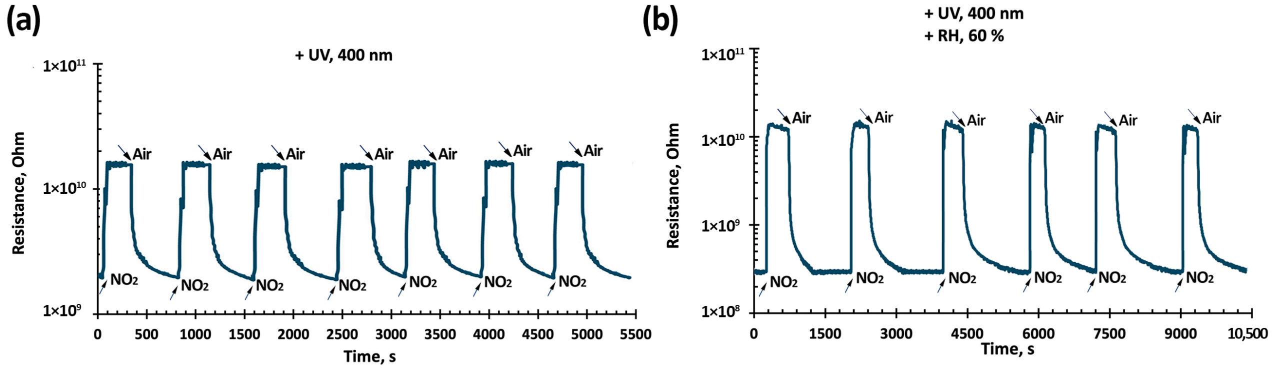

Figure S1. $3TiO_2$–$97SnO_2$ sensors temporal stability (one month after the first measurement) when exposed to 3.85 ppm NO_2 (RT) under light activation (a) and under light activation and 60% RH (b) in different days.
