# Supplementary figures and images for: Excellent Room-Temperature NO2 Gas-Sensing Properties of TiO2-SnO2 Composite Thin Films Under Light Activation
Source: Nanomaterials (Basel). 2025 Jun 5;15(11):871. doi: 10.3390/nano15110871 (PMC12158011; doi:10.3390/nano15110871)

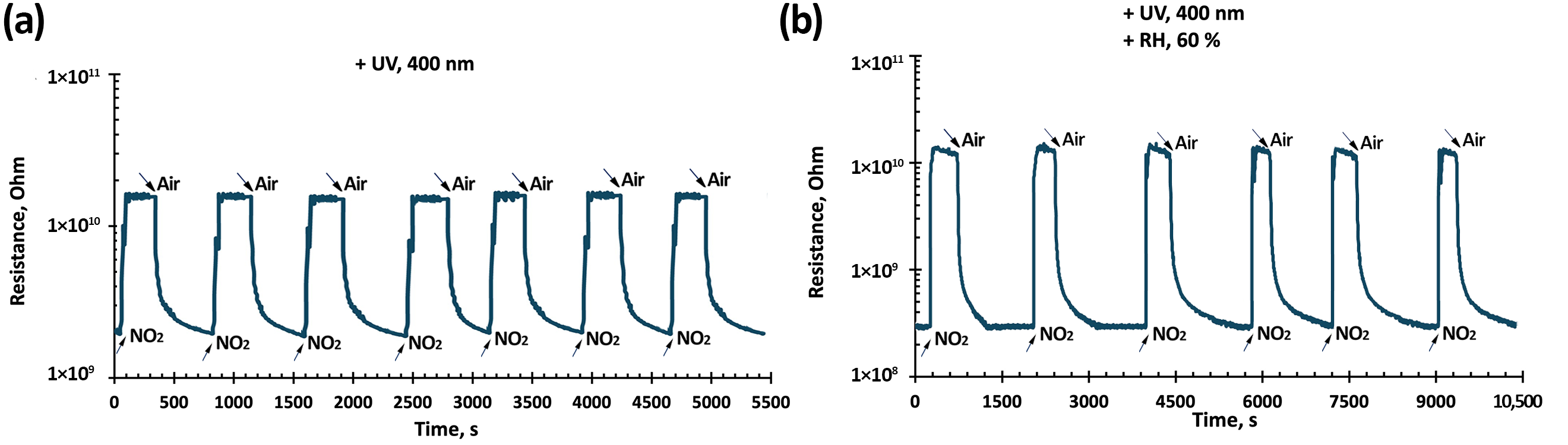

Supplement: Supplementary file 1 [file nanomaterials-15-00871-s001.zip › Fig S1.tif]
